# High Spectrum Efficiency and High Security Radio-Over-Fiber Systems with Compressive-Sensing-Based Chaotic Encryption

**DOI:** 10.3390/mi17010080

**Published:** 2026-01-07

**Authors:** Zhanhong Wang, Lu Zhang, Jiahao Zhang, Oskars Ozolins, Xiaodan Pang, Xianbin Yu

**Affiliations:** 1 College of Information Science and Electronic Engineering, Zhejiang University, Hangzhou 310027, China; zhanhongwang@zju.edu.cn (Z.W.); zhangjh914@zju.edu.cn (J.Z.); xipa@zju.edu.cn (X.P.); 2 Institute of Photonics, Electronics and Telecommunications, Riga Technical University, 1048 Riga, Latvia; oskars.ozolins@rtu.lv

**Keywords:** compressive sensing, radio-over-fiber, chaotic encryption

## Abstract

With the increasing demand for high throughput and ultra-dense small cell deployment in the next-generation communication networks, spectrum resources are becoming increasingly strained. At the same time, the security risks posed by eavesdropping remain a significant concern, particularly due to the broadcast-access property of optical fronthaul networks. To address these challenges, we propose a high-security, high-spectrum efficiency radio-over-fiber (RoF) system in this paper, which leverages compressive sensing (CS)-based algorithms and chaotic encryption. An 8 Gbit/s RoF system is experimentally demonstrated, with 10 km optical fiber transmission and 20 GHz radio frequency (RF) transmission. In our experiment, spectrum efficiency is enhanced by compressing transmission data and reducing the quantization bit requirements, while security is maintained with minimal degradation in signal quality. The system could recover the signal correctly after dequantization with 6-bit fronthaul quantization, achieving a structural similarity index (SSIM) of 0.952 for the legitimate receiver (Bob) at a compression ratio of 0.75. In contrast, the SSIM for the unauthorized receiver (Eve) is only 0.073, highlighting the effectiveness of the proposed security approach.

## 1. Introduction

As the demand for high capacity continues to grow in the next-generation wireless communication networks, high-frequency electromagnetic waves, such as millimeter waves (mmW) and terahertz waves (THz), have garnered significant attention. These frequencies offer broad bandwidth resources, which are crucial for meeting the capacity requirements of future networks [1]. However, their use in radio access networks (RANs) presents several challenges. On one hand, high-frequency communication can significantly enhance the throughput of radio remote units (RRUs), enabling the future 6G network to achieve peak rates of 1 Tb/s and user-experienced data rates of beyond 1 Gb/s [2]. On the other hand, these high-frequency waves experience greater path loss, necessitating ultra-dense deployment of RRUs to maintain coverage and performance [3]. Therefore, improving spectrum efficiency becomes essential for the optical fronthaul networks. Currently, the common public radio interface (CPRI) is widely used for optical fronthaul networks, particularly in Option 8 of the 3GPP function split options [4]. However, CPRI imposes high communication overhead, as it requires up to 15-bit quantization to maintain signal integrity, which results in significant bandwidth consumption and spectrum efficiency degradation [5]. This makes it clear that new methods are needed to improve the spectrum efficiency of optical fronthaul systems.

Several innovative strategies have been proposed to address this challenge. Analog radio-over-fiber (A-RoF) systems, for instance, have been experimentally implemented, as they require less bandwidth for transmission compared to digital signals [6,7]. However, analog signals are more susceptible to the nonlinear effects of optical fibers, necessitating complex nonlinear equalization algorithms that increase the demand for digital signal processing (DSP). Additionally, while data compression algorithms have been suggested to reduce transmission data volume, their effectiveness remains uncertain [8,9]. Digital–analog radio-over-fiber (DA-RoF) systems can enhance the signal-to-noise ratio (SNR) and support high-order modulation formats to improve spectrum efficiency, but they also require digital–analog hybrid transmission and complex signal recovery algorithms, which drive up deployment costs [10]. Alongside these technical challenges, security is a crucial concern for RANs. Due to the broadcast nature of the downlink in optical fronthaul networks, transmitted signals are vulnerable to eavesdropping by unauthorized receivers. In recent years, physical (PHY)-layer encryption techniques have been explored to provide more robust security than traditional media access control (MAC)-layer encryption [11,12]. One promising PHY-layer encryption method is chaotic encryption, which is favored for its high randomness and low implementation cost [13,14,15,16]. Recently, a CS-based chaotic encryption algorithm was proposed to improve the security performance of the communication system, embedding the chaotic encryption algorithm into the CS algorithm [17,18]. In addition, the performance of the spectrum efficiency is not discussed in these works.

In this paper, we present an approach that combines high spectrum efficiency with robust security by using the compressive sensing (CS)-based chaotic encryption algorithm in a radio-over-fiber (RoF) system. We generated a 16QAM-DMT signal and mapped it to an 8 Gbit/s BPSK signal via fronthaul quantization and transmitted it over 10 km of standard single-mode fiber (SSMF) at the C-band. A photonics-based method is used to generate the RF signal at 20 GHz. With the CS-based chaotic encryption algorithm, the system achieves quantization-error-free at a 6-bit quantization level, even with compression ratios (CR) of 0.25 and 0.5. Furthermore, the system’s security is ensured, as demonstrated by the structural similarity index (SSIM) of 0.952 for the legitimate receiver (Bob) at a compression ratio of 0.75, indicating minimal loss of information. In contrast, the SSIM for the eavesdropper (Eve) is only 0.073 under the same conditions, highlighting the effectiveness of the proposed security method.

The remainder of the paper is organized as follows: Section 2 introduces the selected chaotic sequence and the principle of the algorithms. Section 3 explains the experiment setup for the 20 GHz RoF system, and Section 4 discusses the experiment results and optimizes the parameters of the algorithm. Finally, Section 5 concludes the paper with a summary of the research.

## 2. Principle

**Figure 1:**
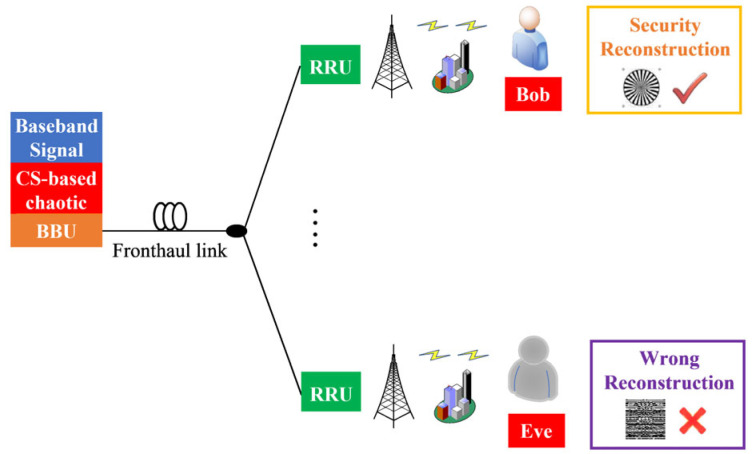
The architecture of the high-spectrum-efficiency and high-security RoF system.

The architecture of the proposed RoF system is shown in Figure 1. The data is processed by the CS-based chaotic encryption algorithm at the BBU and then transmitted to multiple RRUs. At the RRUs, RF signals are generated and sent to the users. First, the amount of transmitted data is reduced, and a lesser quantization bit number will be used for the optical fronthaul network because of the CS algorithm. On top of that, because of the chaotic encryption, the legal receiver Bob can reconstruct the origin signal with minimal quality degradation, and the illegal receiver Eve obtains less effective information due to the absence of the legal keys. Therefore, high spectrum efficiency and high security can be achieved in the proposed system.

**Figure 2:**
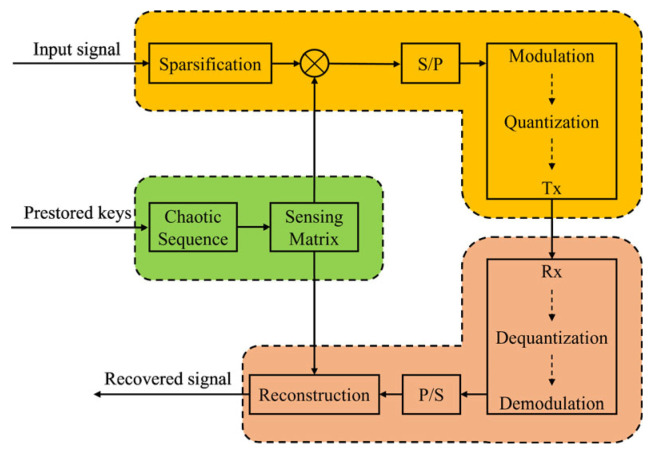
Schematic for the compressive-sensing-based chaotic encryption algorithm.

A generalized algorithm block diagram is shown in Figure 2. The algorithm will be discussed in detail. First, the key is prestored in the BBU and the legal receivers. With the key, the chaotic sequence can be generated at the BBU. In the proposed system, the 2-dimensional logistic-sine-coupling map (2D-LSCM) is chosen as the chaotic sequence, which can be expressed as

(1) $$\left\{ \begin{array}{l} x_{i+1}=\sin(\pi (4\theta x_{i}(1-x_{i})+(1-\theta )\sin(\pi y_{i}))\\ y_{i+1}=\sin(\pi (4\theta x_{i}(1-x_{i})+(1-\theta )\sin(\pi x_{i+1})) \end{array}\right..$$

Here, *x*_1_, *y*_1_ and *θ* are chosen as the keys, where θ ∈ [0, 1], x_1_, y_1_∈(0, 1). Compared to other sequences, such as the Logistic map and the Lorenz system, 2D-LSCM exhibits hyperchaotic properties over a larger range, with better chaotic performance and lower computational complexity [19]. Then utilizing the keys, the sensing matrix **B** for the compressive sensing can be obtained. According to mathematical theory, the matrix **B** needs to meet the restricted isometry property (RIP) condition for signal reconstruction at the receiver. The random circular matrix (RCM) is utilized in our system to meet the condition because of its simplicity [20]. The cyclic characteristic of RCM is

(2) $$\begin{array}{l} B(j,1)=\lambda \cdot B(j-1,n)\\ B(j,2:n)=\lambda \cdot B(j-1,1:n-1), \end{array}$$

where 2 ≤ *j* ≤ m, *λ* > 1. The first line of **B** can be set as the corresponding number of the average of *x_n_* and *y_n_*. For the input transmitted signal, it needs to be sparsified by orthonormal basis decomposition due to the condition for compressive sensing. For example, an *n* × 1 vector **x** can be decomposed as

(3) $$x=\sum_{k=1}^{n}\psi_{k}s_{k}=\psi s,$$

where **ψ** is the orthonormal basis of **x**, and **s** is an *n* × 1 sparse vector. For multimedia signals such as images, the frequency domain matrix is sparse because of low high-frequency components, and the sparsification can be completed by time-frequency transform directly, such as Fast Fourier Transform (FFT), Discrete Cosine Transform (DCT), and Discrete Wavelet Transform (DWT). Among them, the range of matrix elements obtained after DCT can be represented using only 14 binary digits, which is smaller than the 18 bits of DWT and the 31 bits of FFT [17]. Therefore, in algorithm design, DCT is adopted for sparsification to reduce data volume. Then the vector **x** is multiplied by an *m* × *n* measurement matrix **Φ** for low-dimensional mapping, which can be expressed as

(4) y=Φx+e=Φψs+e=Bs+e,

where **B** is the *m* × *n* sensing matrix obtained by a chaotic sequence. Here **y** is an *m* × 1 matrix, and the CR is defined as *m*/*n*. Then after parallel serial conversion, the signal is modulated and quantized by the BBU and then transmitted to RRU for frequency conversion.

At the legal receiver, the keys are prestored and the chaotic sensing matrix **B** can be cloned. In fact, the problem of solving **s** can be transformed into an optimization problem [21], which is

(5) $$\begin{array}{l} \min||s|{|}_{0}\\ \mathrm{subject}\;\mathrm{to}\;||y-Bs|{|}_{2}\leq \varepsilon_{e}, \end{array}$$

where *ε_e_* is the energy of the sampling noise. It is difficult to solve the NP-hard problem by an analytical method. The smooth *l*_0_ method can be used in this scene [22]. This method uses a Gaussian function to fit the *l*_0_-norm of vector **s**, converting it into a smooth continuous function for optimization. The Gaussian function is

(6) $$f_{\sigma }(s_{i})=1-\exp(-\frac{s_{i}^{2}}{2\sigma^{2}}),$$

where σ is the smoothness factor. Then the *l*_0_-norm of vector **s** is

(7) $$\|s{\|}_{0}=\underset{\sigma \to \infty }{\lim}F_{\sigma }(s_{i})=\underset{\sigma \to \infty }{\lim}(n-\sum_{i=1}^{n}f_{\sigma }(s_{i})).$$

Because of the smooth, continuous characteristic, gradient descent can be utilized to find the minimum point of *l*_0_-norm. The initial vector is the multiplication of the pseudo-inverse of the matrix **B** and the measurement vector **y**. After several iterations, the algorithm convergence is achieved, and the sparsest solution of the problem in Equation (5) is obtained. However, because of a lack of knowledge of the keys, the chaotic sensing matrix cannot be cloned correctly at the illegal receiver Eve. Therefore, Eve can obtain less effective information, and the security of the RoF system is ensured.

## 3. Experimental Setup

**Figure 3:**
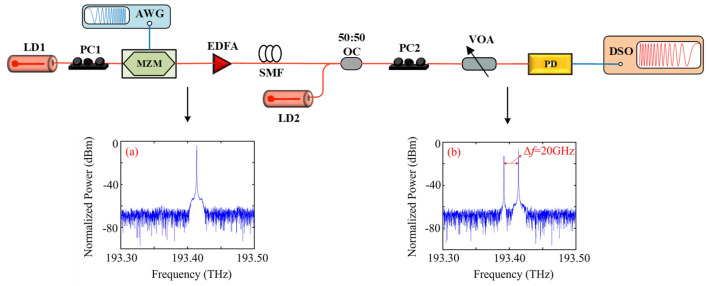
Experimental setup of the proposed RoF system. Inset: (**a**) the normalized optical spectrum after MZM, (**b**) the normalized optical spectrum before PD.

The setup for the proof-of-concept experiment is illustrated in Figure 3. A 64 × 64 image signal, labeled ‘testpat1’, is chosen as the input signal. First, the image signal undergoes a sparse transformation using DCT, followed by processing with the CS-based chaotic encryption algorithm. The resulting compressed signal is then modulated into a 16-QAM signal. Next, the signal is converted into a DMT signal through subcarrier mapping and IFFT, with 2048 IFFT points. Of these, 800 subcarriers are used to carry the 16-QAM signal, while another 800 subcarriers carry the complex conjugate of the 16-QAM signal. The DMT signal is subsequently quantized and remapped to a BPSK signal for optical fronthaul transmission.

For the optical transmission, intensity modulation is employed. The output wavelength of laser diode LD1 (100 kHz linewidth) is set to 1500.00 nm, and the laser is modulated by a Mach-Zehnder Modulator (MZM, EOSpace, 40-GHz bandwidth). An 8 Gbps total signal is generated by an arbitrary waveform generator (AWG, Keysight M8195A, 64 GSa/s) to drive the MZM. The modulated laser is then amplified by an erbium-doped fiber amplifier (EDFA) to increase the power budget. After traveling through 10 km standard single-mode fiber (SSMF), the signal is coupled with a local oscillator (LO) laser from LD2 (100 kHz linewidth), which operates at a wavelength of 1550.16 nm, using a 50:50 coupler. A variable optical attenuator (VOA) is used to control the input power to the photodiode (PD, 40 GHz bandwidth). In the PD, the 20 GHz RF signal is generated via photo-mixing and transmitted through an RF cable. The RF signal is captured by a digital storage oscilloscope (DSO, Keysight DSOX93204A, 80 GSa/s) for offline DSP.

The signal recovery process follows these steps in the DSP flow. First, the signal is resampled at 64 GSa/s from DSO and converted to baseband through digital down-conversion. Due to the free-running characteristics of the two laser diodes, frequency offset (FO) can impact communication performance. The FO is estimated using a digital phase-locked loop [23], and synchronization is achieved via a cross-correlation algorithm. To compensate for dispersion, a 4-tap feedforward equalizer is employed, followed by a decision feedback equalizer with four feedforward taps and three feedback taps [24]. After equalization, the BPSK signal is recovered, and dequantization and demodulation are performed to retrieve the compressed signal. The sparsest solution to the optimization problem is found using the greedy descent algorithm, with 40 iterations and a step size of 1.01. The original signal is then reconstructed through IDCT.

## 4. Results and Discussion

**Figure 4:**
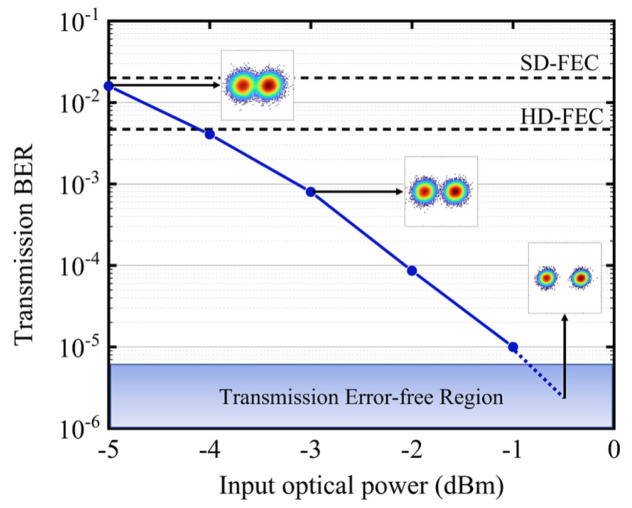
The transmission performance of the proposed RoF system.

The transmission performance of the proposed RoF system is shown in Figure 4. The bit error rate (BER) is evaluated by comparing the transmitted and received BPSK signals. Given the non-Gaussian distribution of the actual transmission signal, the robustness of the system is critical. The results show that the BER improves with increasing optical power at the photodiode (PD). The system maintains a BER below the hard-decision forward error correction (HD-FEC) threshold of 3.8 × 10^−3^ [25] when the optical power exceeds −4 dBm, and error-free transmission is achieved when the optical power exceeds 0 dBm. The BPSK constellation diagram in Figure 3 further demonstrates the performance.

The spectrum efficiency of the system is discussed. To ensure a fair comparison, spectral efficiency is evaluated under two conditions: one using the CS-based chaotic encryption algorithm and the other with traditional encryption but without CS. In both cases, the input power to the photodiode (PD) is set to 5 dBm to achieve error-free transmission. With the CS-based algorithm, the amount of transmitted data is significantly reduced. For instance, when the CR is 0.5, the number of symbols per subcarrier in a frame decreases from 20 to 9. This reduction allows the system to transmit twice the amount of data in the same transmission bandwidth. Similarly, when the CR is 0.25, nearly four times the amount of data can be transmitted. However, it is important to note that a lower CR can lead to greater signal degradation during reconstruction. This represents a trade-off between spectrum efficiency and signal quality.

**Figure 5:**
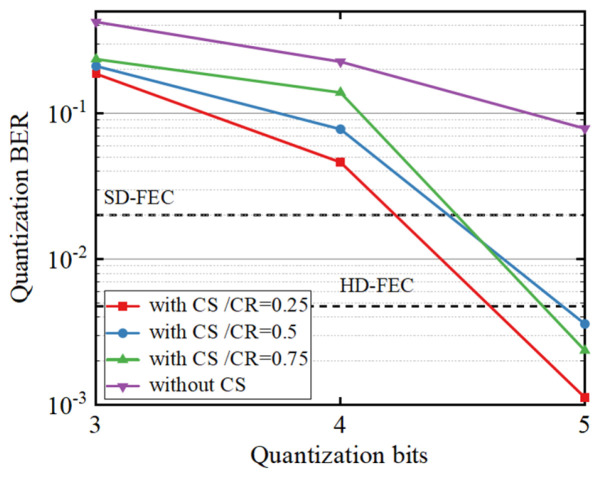
The quantization BER performance under different fronthaul quantization bit numbers when error-free transmission is achieved.

The effect of fronthaul quantization is also examined in our experiment. Figure 5 shows the BER performance of the system with different CR values. Since the system achieves error-free transmission, the focus is on estimating the effect of transmission errors. With the CS algorithm, the quantization BER improves compared to the case without CS, meaning that fewer quantization bits are required in the RoF system. As a result, the system’s spectrum efficiency is further enhanced. This improvement is because the CS algorithm reduces signal redundancy and the amplitude of the DMT signal [26], allowing the signal to be represented with fewer quantization bits. However, the quantization BER degrades as the CR increases since lower CR values eliminate more redundant information from the sparse signal. Notably, error-free quantization can be achieved when the CR is 0.25 or 0.5, demonstrating the effectiveness of the CS-based approach in reducing the required quantization bits while maintaining signal integrity.

**Figure 6:**
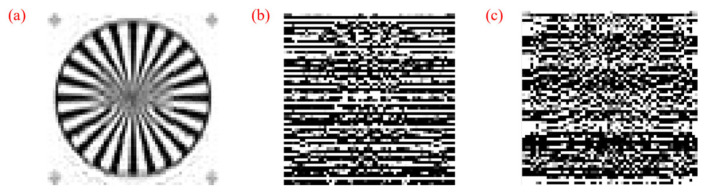
Images recovered by (**a**) Bob and (**b**) Eve with incorrect keys (*x*_1_, *y*_1_, *θ*) = (0.7, 0.7, 0.8) and (**c**) Eve with incorrect keys (*x*_1_, *y*_1_, *θ*) = (0.6 + 10^−14^, 0.7, 0.8) when CR = 0.5.

Then the security of the system is verified. The SSIM performance and the quality of the reconstructed images are shown in Figure 6 and Table 1. The legal keys (*x*_1_ = 0.6, *y*_1_ = 0.7, *θ* = 0.8) are prestored in Bob (*x*_1_ = 0.7, *y*_1_ = 0.7, *θ* = 0.8), and there are only incorrect keys in Eve. As a result, Bob, the legitimate receiver, can successfully reconstruct the image using the correct decryption keys, while Eve, the unauthorized receiver, cannot. When the CR is 0.75, the SSIM between Alice and Bob reaches 0.952, indicating only minimal quality degradation due to the CS-based chaotic encryption algorithm. In contrast, the SSIM between Alice and Eve remains close to 0, meaning that Eve is unable to extract meaningful information from the reconstructed image. Then the situation of perturbation is also considered. Here, the disturbance is set to 10^−14^, and the SSIM of Eve is 0.033 with CR = 0.5 and 0.028 with CR = 0.75, confirming the sensitivity to the keys. With the sensitivity of the chaotic sequency is 10^−14^, the key space can be calculated as (10^14^)^3^ = 10^42^, which is sufficient to prevent attacks from Eve. This demonstrates that the CS-based chaotic encryption algorithm effectively ensures the security of the RoF system, preventing unauthorized access.

**Figure 7:**
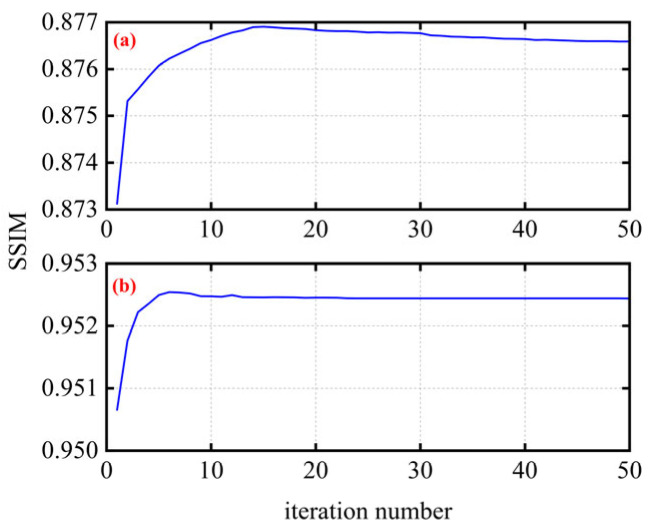
The SSIM performance under different iteration numbers of the smooth *l*_0_ method with (**a**) CR = 0.5 and (**b**) CR = 0.75.

The optimized parameters for the smooth *l*_0_ method are also discussed. The SSIM performance of Bob with different iteration numbers is illustrated in Figure 7. For iteration numbers, the step size is set as 1.01, and the CR is set as 0.5. From the results, we found that there is not much difference in SSIM performance under different iteration times. When the iteration number is small, SSIM performance has slightly improved with its improvement. Afterwards, the algorithm converges and SSIM performance gradually stabilizes. Since the improvement is not very significant, the iteration number can be reduced to lower the algorithm complexity and meet real-time communication requirements. In fact, the complexity of the compressive sensing algorithm is approximately *O*(*N*), which is completely acceptable for real-time communication [17]. Based on existing deductions, the initial smooth factor for the compressive sensing algorithm may be chosen about two to four times the maximum absolute value of the obtained sources. In the proof-of-concept experiment, we set the factor to two times the maximum absolute value to ensure mathematical interpretability.

**Figure 8:**
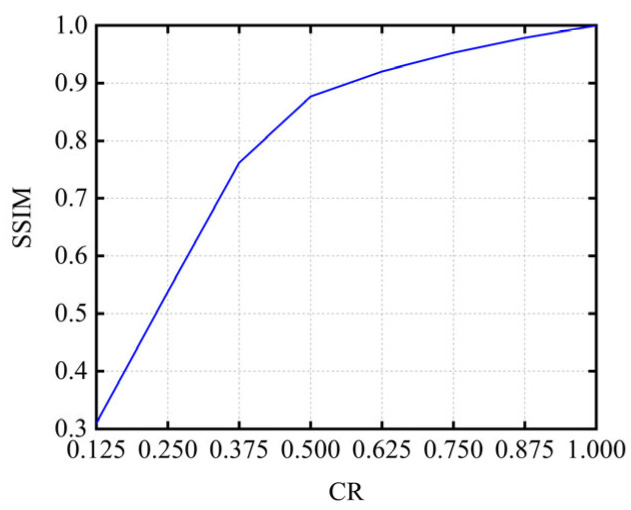
The SSIM between Alice and Bob under different CR conditions.

The selection of CR also has a significant impact on the signal reconstruction performance of Bob. The SSIM performance of Bob under different CRs is shown in Figure 8. Clearly, when the CR is less than 0.5, the SSIM performance significantly decreases. There are two main reasons for this result. Firstly, the DCT of the image signal is not a strictly sparse matrix, and there are still small coefficients at high frequencies. When the number of samples is reduced, the effective sparsity of the matrix remains unchanged, making it difficult for the algorithm to distinguish between the main components and noise based on redundant information. On the other hand, the observation process of CS is accompanied by noise. When the CR decreases, the number of elements in the matrix also decreases. Due to the diffusion effect of matrix multiplication, the SNR of each sampling point decreases. Therefore, to ensure the signal quality reconstructed by Bob, it is best to choose a CR greater than 0.5. And adaptive adjustment algorithms can be further adopted to balance the computational complexity and signal reconstruction quality.

## 5. Conclusions

In this paper, we propose a novel method that utilizes a CS-based chaotic encryption algorithm to enhance both spectrum efficiency and security of the RoF system. Regarding spectrum efficiency, the CS algorithm reduces the amount of transmitted data, and the system achieves quantization-error-free at only a 6-bit quantization level with the proposed algorithm. In terms of security, an SSIM of 0.952 for Bob at a CR of 0.75 is achieved, indicating minimal information loss. In contrast, under the same condition, the SSIM for Eve is only 0.073, confirming the effectiveness of the algorithm. On this basis, the parameters are optimized and explored to further reduce computational complexity.

## Figures and Tables

**Table 1:** The SSIM between the image transmitted and the one recovered with 6-bit quantization.

Receiver	CR = 0.5	CR = 0.75
Bob	0.877	0.952
Eve [(*x*_1_, *y*_1_, *θ*) = (0.7, 0.7, 0.8)]	0.052	0.073
Eve [(*x*_1_, *y*_1_, *θ*) = (0.6 + 10^−14^, 0.7, 0.8)]	0.033	0.028

## Data Availability

Dataset available on request from the authors.
